# A prospective multicenter clinical research study validating the effectiveness and safety of a chest X-ray-based pulmonary tuberculosis screening software JF CXR-1 built on a convolutional neural network algorithm

**DOI:** 10.3389/fmed.2023.1195451

**Published:** 2023-08-15

**Authors:** Yang Yang, Lu Xia, Ping Liu, Fuping Yang, Yuqing Wu, Hongqiu Pan, Dailun Hou, Ning Liu, Shuihua Lu

**Affiliations:** ^1^Department of Tuberculosis, Shanghai Public Health Clinical Center Affiliated to Fudan University, Shanghai, China; ^2^Department of Pulmonary Medicine, National Clinical Research Center for Infectious Disease, Shenzhen Third People's Hospital/The Second Affiliated Hospital, School of Medicine, Southern University of Science and Technology, Shenzhen, Guangdong, China; ^3^Department of Tuberculosis, Chongqing Public Health Medical Center, Southwest University, Chongqing, China; ^4^Department of Tuberculosis, Jiangxi Chest Hospital, Nanchang, Jiangxi, China; ^5^Department of Tuberculosis, The Third Hospital of Zhenjiang, Zhenjiang, Jiangsu, China; ^6^Department of Radiology, Beijing Chest Hospital, Capital Medical University, Beijing, China; ^7^Department of Tuberculosis, Hebei Chest Hospital, Shijiangzhuang, Hebei, China

**Keywords:** tuberculosis, screening, radiography, deep learning, convolutional neural network algorithm, computer-aided detection (CAD), diagnosis

## Abstract

**Background:**

Chest radiography (chest X-ray or CXR) plays an important role in the early detection of active pulmonary tuberculosis (TB). In areas with a high TB burden that require urgent screening, there is often a shortage of radiologists available to interpret the X-ray results. Computer-aided detection (CAD) software employed with artificial intelligence (AI) systems may have the potential to solve this problem.

**Objective:**

We validated the effectiveness and safety of pulmonary tuberculosis imaging screening software that is based on a convolutional neural network algorithm.

**Methods:**

We conducted prospective multicenter clinical research to validate the performance of pulmonary tuberculosis imaging screening software (JF CXR-1). Volunteers under the age of 15 years, both with or without suspicion of pulmonary tuberculosis, were recruited for CXR photography. The software reported a probability score of TB for each participant. The results were compared with those reported by radiologists. We measured sensitivity, specificity, consistency rate, and the area under the receiver operating characteristic curves (AUC) for the diagnosis of tuberculosis. Besides, adverse events (AE) and severe adverse events (SAE) were also evaluated.

**Results:**

The clinical research was conducted in six general infectious disease hospitals across China. A total of 1,165 participants were enrolled, and 1,161 were enrolled in the full analysis set (FAS). Men accounted for 60.0% (697/1,161). Compared to the results from radiologists on the board, the software showed a sensitivity of 94.2% (95% CI: 92.0–95.8%) and a specificity of 91.2% (95% CI: 88.5–93.2%). The consistency rate was 92.7% (91.1–94.1%), with a Kappa value of 0.854 (*P* = 0.000). The AUC was 0.98. In the safety set (SS), which consisted of 1,161 participants, 0.3% (3/1,161) had AEs that were not related to the software, and no severe AEs were observed.

**Conclusion:**

The software for tuberculosis screening based on a convolutional neural network algorithm is effective and safe. It is a potential candidate for solving tuberculosis screening problems in areas lacking radiologists with a high TB burden.

## Introduction

Tuberculosis (TB) remains one of the leading causes of death worldwide, killing 1.4 million people per year (1). Although the disease is largely curable and preventable, an estimated 2.9 million per 10 million people falling sick with TB were not diagnosed or reported to the World Health Organization (WHO) (2). Therefore, there is a pressing need to improve the early diagnosis of TB disease, thus initiating treatment promptly and reducing the transmission of *Mycobacterium tuberculosis*. One effective strategy is systemic screening, which should distinguish people with a high possibility of TB from those without. Chest radiography (chest X-ray or CXR) is a widely used and cost-effective screening tool for TB detection, achieving a sensitivity and specificity of 85.0 and 96.0% for TB-related abnormalities, respectively (2–5). While in areas with low resources and a high TB burden requiring mass screening urgently, the interpretation of CXRs is labor-intensive and time-consuming under the circumstances that trained health personnel interpreting the CXRs are very lacking (6–11).

Computer-aided detection (CAD) technologies, especially those with AI algorithms, vastly increase the capacity of image reading and have similar or even better diagnostic accuracy performance compared to human readers (12–14). The technologies make it possible to perform accurate mass screening with fewer resources. Thus, the WHO has recommended CAD as an alternative to human interpretation of CXR for screening and triaging pulmonary TB in individuals aged 15 years or older (2). The edge of CAD is artificial intelligence, especially in the field of deep learning, in which convolutional neural networks (CNNs) are the most promising algorithms for dealing with visual tasks (15–17). JF CXR-1 (version 2) (JF Healthcare, Jiangxi, China) is a simultaneous CXR detection CAD software based on CNNs that detects multiple thorax diseases, such as TB, lung mass, and lung nodules. The software was trained on 14,160 CXRs from township-level hospitals across China. In the testing phase, 13122 CXRs were provided for JF CXR-1 to detect TB. Among them, 31.5% (4127/13122) were pulmonary tuberculosis, 16.3% (2143/13122) were other pulmonary diseases such as pneumonia, pulmonary abscess, lung cancers, etc., and 52.2% (6852/13122) were normal. And JF CXR-1 has achieved an AUC of 0.94, a sensitivity of 0.91 (3,755/4,127), and a specificity of 0.81 (7,286/8,995), which meet the WHO's criteria for the target product profile. To validate its effectiveness and safety in clinical use, we conducted prospective multicenter clinical research in six general infectious disease hospitals in mainland China.

## Methods

### Study setting and population

The study was conducted at Shanghai Public Health Clinical Center, Beijing Chest Hospital, the Third Hospital of Zhenjiang, Chongqing Public Health Medical Center, Jiangxi Province Chest Hospital, and Hebei Chest Hospital. All of them are designated hospitals for tuberculosis in China, and there are also healthy individuals in the medical examination centers of each hospital. Participants were recruited from the visitors from tuberculosis clinics and medical examination centers of the above hospitals since June 2020. The inclusion criteria were (1) being aged 15 or over, (2) being willing to receive the CXR examination or could provide the image (DICOM format) of the posterior-anterior CXR taken in the late 40 days, and (3) voluntarily participating and providing informed written consent. The exclusion criteria included (1) a history of obsolete pulmonary tuberculosis, (2) neutropenia, (3) infection with the human immunodeficiency virus (HIV), (4) subjects whose CXR images do not meet the diagnostic requirements, (5) those with a history of hematological disorders, (6) those with a history of pulmonary lobectomy, (7) those with a history of mental illness or cognitive disorder, (8) those who are pregnant or breastfeeding, (9) those who had participated in pharmaceutical clinical research within 30 days, and (10) those who had other conditions that investigators consider inappropriate for research participation. All enrolled participants provided informed written consent. This study was approved by the ethics committees of Shanghai Public Health Clinical Center, Beijing Chest Hospital, The Third Hospital of Zhenjiang, Chongqing Public Health Medical Center, Jiangxi Province Chest Hospital, and Hebei Chest Hospital, respectively.

### Procedures

Each participant received a complete blood count (CBC) test and an HIV antibody test. Participants with neutropenia or/and HIV infection were excluded according to the results of the blood tests. Then, each of the rest of the participants received a digital posterior-anterior CXR. Even though not all the centers have the same X-ray machines, efforts were made to set the parameters as similar as possible. The CXR images were saved in DICOM format and imported into the AI-based CAD software (JF CXR-1 v2, produced by Jiangxi Zhongke Jiufeng Smart Medical Technology Co., Ltd.). JF-CXR-1 and the radiologist group read every single CXR image independently and were blinded to all the information, including age and sex. The AI algorithm would produce a probability score for each anonymized image to predict its likelihood of being TB-positive.

A score >0.35 indicated a high possibility of tuberculosis, prompting the need for further diagnostic measures such as microbiological tests and a chest CT scan (When the threshold score = 0.35, the combination of sensitivity, specificity, and Kappa value is the best). The radiologist group consisted of eight certified senior radiologists from a third-party organization. They utilized the Diagnosis for Pulmonary Tuberculosis of Chinese Health Industry Standards as the fundamental criteria for TB x-ray screening. All radiologists have at least 10 years of experience in Grade-A tertiary hospitals. The CXR images were read independently by five senior radiologists. Diagnosis suggestions in CXR reports with “suspect TB” or “TB” were considered positive for TB. “Normal” and “other abnormal” CXR were considered negative for TB. The final decision among the five radiologists was determined based on the principle that the minority is subordinate to the majority. If cases where three radiologists shared the same opinion but the other two disagreed, the image would be sent to three other radiologists for arbitration. The final decision of the radiologist group was determined by the three “arbitrators” in a written report. Two months after the CXR examination, the clinical diagnosis information of every participant was collected. TB diagnosis followed China's National TB Diagnosis Guideline (WS288-2017).

### Data analysis

All the data were statistically described, including baseline information, effectiveness data, and safety data. The final decision of the radiologist group was set as the reference standard for the software to compare. For effectiveness, the main evaluation indicators were sensitivity and specificity, referring to the results of the radiologists' board. The secondary evaluation indicators were the area under the receiver operating characteristic (ROC) curve (AUC) and the consistency rate with the final diagnosis (including bacteriologically confirmed and clinical diagnoses). If an image was diagnosed as TB or non-TB by both the radiologist group and the AI software, it was defined as true positive (TP) or true negative (TN), respectively. If an image was diagnosed as TB by the radiologist group but non-TB by the software, it was defined as a false negative (FN). Moreover, if an image was diagnosed as non-TB by the radiologist group but TB by the software, it was defined as a false positive (FP). Sensitivity = TP/(TP + FN), specificity = TN/(TN + FP) and the consistency rate = (TP + TN)/(TP + TN + FP + FN). The ROC curve was acquired when the sensitivity was set as the *Y*-axis, and 1-specificity was set as the *X*-axis. And the AUC was the area under the ROC.

Sensitivity is the proportion of true positive tests in all patients with a condition. A test or instrument can yield a positive result for a subject with that condition. In our study, it means the test ability of AI software screening out TB on the CXRs compared with the radiologist group.

Specificity is the percentage of people without the disease who are correctly excluded by the test. It is important to exclude people with diseases during screening. In our study, it refers to the ability of AI software to rule out non-TB participants. Ideally, a test should provide high sensitivity and specificity. For safety, adverse events (AE) and severe adverse events (SAE) were evaluated for every participant since their CXR was imported into the AI software and stopped 2 weeks later.

### Role of the AI developers

The AI developer had no role in study design, data collection, analysis, or manuscript writing, but they provided us with a free account to use the software and free technical support.

## Results

**Figure 1:**
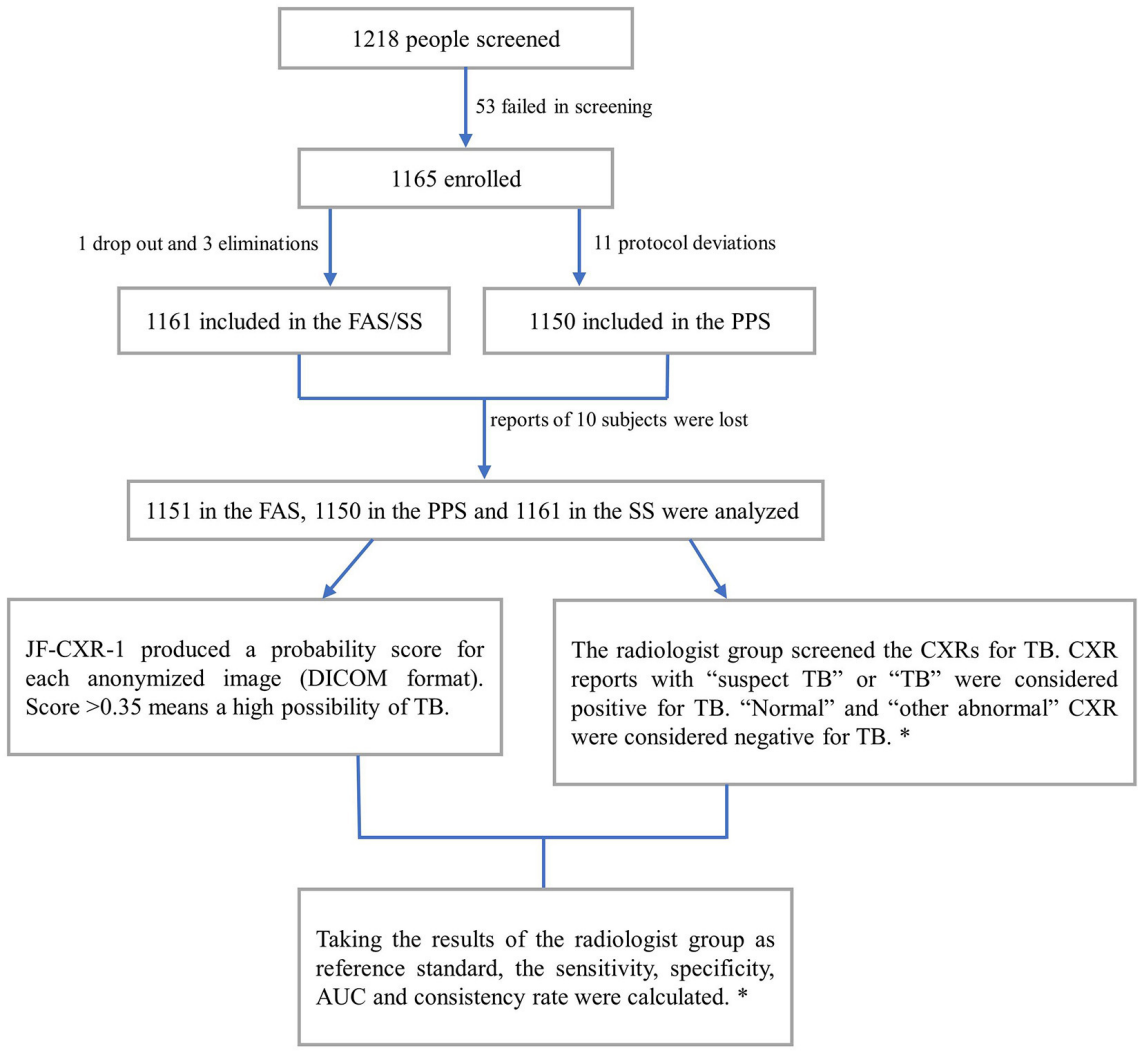
Participant selection process and the diagnostic workflow. FAS, analysis set; SS, safety analysis set; PPS, per protocol set; AUC, area under the receiver operating characteristic curves. *, details are in the methods section.

Between 06 Nov 2020, and 04 Jun 2021, 1,218 participants were screened, and 53 failed screening. Thus, 1,165 were enrolled, 1,161 were included in the Full Analysis Set (FAS) and Safety Analysis Set (SS) due to one dropout and three eliminations, and 1,150 were included in the Per Protocol Set (PPS) due to 11 protocol deviations (Table 1; Figure 1). The participants' ages ranged from 15 to 86 years, and the average was 44.4 ± 16.4 years. Men accounted for 60.0% (697/1,161), while women accounted for 40.0% (464/1,161).

**Table 1:** Participants distribution.

**Description**	**Center 01**	**Center 02**	**Center 03**	**Center 04**	**Center 05**	**Center 06**	**Total**
Screening	199	70	154	375	360	60	1,218
Enrollment	186	68	150	358	343	60	1,165
Drop out	0	1	0	0	0	0	1
Elimination	0	0	0	3	0	0	3
Protocol deviation	0	0	0	6	5	0	11
FAS	186	67	150	355	343	60	1,161^*^
PPS	186	67	150	349	338	60	1,150
SS	186	67	150	355	343	60	1,161

Center 01, Shanghai Public Health Clinical Center; Center 02, Beijing Chest Hospital; Center 03, The Third Hospital of Zhenjiang; Center 04, Chongqing Public Health Medical Center; Center 05, Jiangxi Province Chest Hospital; Center 06, Hebei Chest Hospital. FAS, full analysis set; PPS, per protocol set; SS, safety set.

* The reports of ten subjects were lost during an office movement due to the COVID-19.

**Figure 2:**
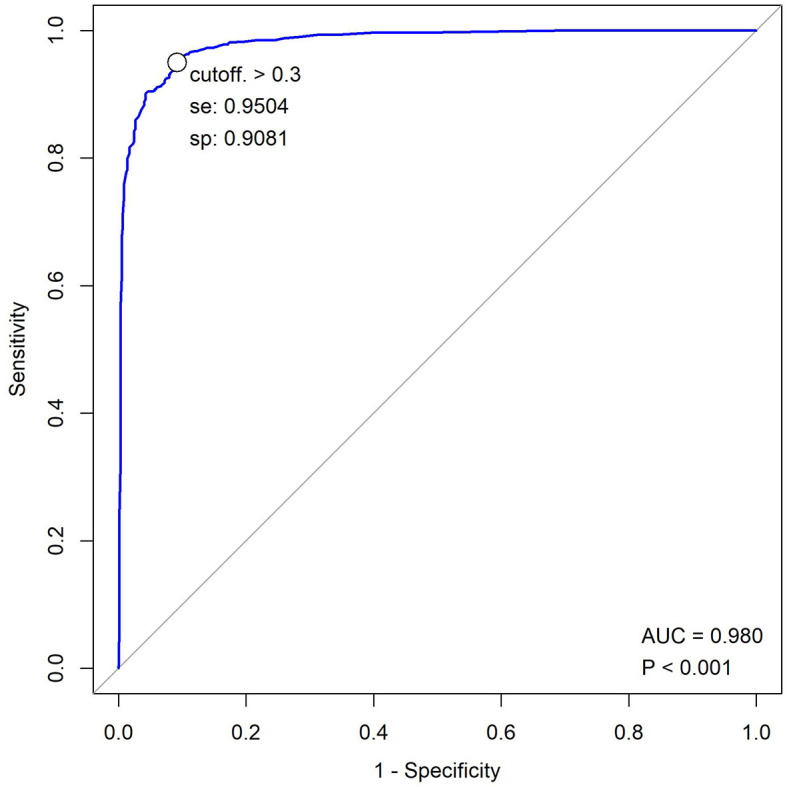
ROC graph for JF CXR-1 of FAS.

In the FAS, the reports of 10 subjects were lost. Thus, the results of 1,151 subjects were analyzed. According to the radiologist group, 601 images were positive for TB, while 550 were negative for TB. Compared with the reference standard, the sensitivity of the software was 94.2% (95% CI: 92.0–95.8%), while the specificity was 91.2% (95% CI: 88.5–93.2%). Therefore, the consistency rate was 92.7% (95% CI: 91.1–94.1%) with a Kappa value of 0.854 (*P* = 0.000) (Table 2). The AUC was 0.98 (Figure 2). After 2 months of clinical evaluation since enrollment, the diagnosis remained unclear for 29 out of 1,161 participants. Among the rest of the 1,132 participants, 687 were diagnosed with tuberculosis, while the others were not. Therefore, taking the final diagnosis (including bacteriologically confirmed and clinically diagnosed) as the ground truth, the sensitivity of the software was 78.9% (75.7–81.8%), the specificity was 89.9% (86.7–92.4%), and the consistency rate was 83.2% (80.9–85.3%) with a Kappa value of 0.662 (*p* = 0.000) (Table 3).

**Table 2:** Performance of JF CXR-1 against radiologists (FAS).^*^

**Results from JF CXR-1**	**Results from radiologists**		**Total sum**
	**TB (+)**	**TB (–)**	
TB (+)	551	50	601
TB (**–**)	34	516	550
Total sum	585	566	1,151
Sensitivity (95%CI)	94.2% (92.0–95.8%)
Specificity (95%CI)	91.2% (88.5–93.2%)
Consistency rate (95%CI)	92.7% (91.1–94.1%)
Kappa	0.854 (*P* = 0.000)

* In the FAS, 10 subjects have no reports of CXR images because they met the exclusion criteria but were mistakenly enrolled.

**Table 3:** Performance of JF CXR-1 against final diagnosis (FAS).^*^

**Results from JF CXR-1**	**Results from clinical diagnosis**		**Total sum**
	**TB (+)**	**TB (–)**	
TB (+)	542	45	587
TB (**–**)	145	400	545
Total sum	687	445	1,132
Sensitivity (95%CI)	78.9% (75.7–81.8%)
Specificity (95%CI)	89.9% (86.7–92.4%)
consistency rate (95%CI)	83.2% (81.0–85.3%)
Kappa	0.662 (*P* = 0.000)

* Final diagnosis included bacteriologically confirmed and clinical diagnosed cases.

**Figure 3:**
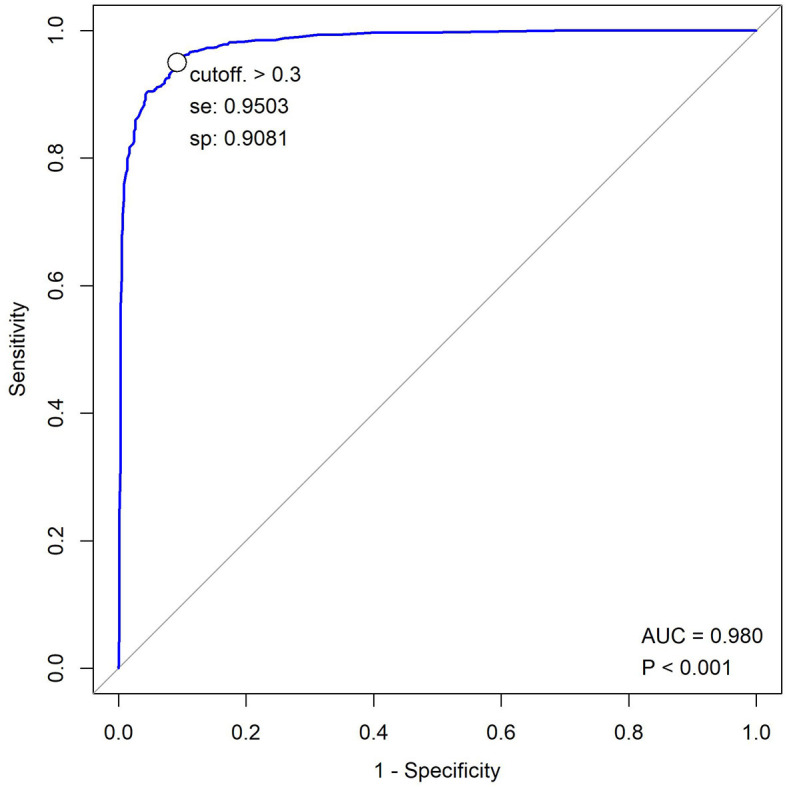
ROC graph for JF CXR-1 of PPS.

In the PPS, the results of 1,150 subjects were analyzed. We found that 600 images were positive for TB, while 550 were negative for TB. Compared with the reference standard, the sensitivity of the software was 94.2% (92.0%−95.8%), while the specificity was 91.2% (88.5–93.2%). Therefore, the consistency rate was 92.7% (91.1–94.1%) with a Kappa value of 0.854 (*P* = 0.000) (Table 4). The AUC was 0.98 (Figure 3). The diagnosis remained unclear for 29 out of 1,161 participants after 2 months of clinical evaluation since enrollment. Among the rest of the 1,121 participants, 582 were diagnosed with tuberculosis, while the other 539 were not. Therefore, taking the final diagnosis (including bacteriologically confirmed and clinically diagnosed) as the ground truth, the sensitivity of the software was 79.3% (76.1–82.2%), the specificity was 90.5% (87.4–92.9%), and the consistency rate was 83.7% (81.4–85.7%) with a Kappa value of 0.671 (*p* = 0.000) (Table 5). Since it has already been reported that the JF CXR-1 v2 algorithm performed worse among older age groups (>60 years) (13), performance analysis of the tool with both >60 and < 60 years was checked parallelly. In the FAS, 20.2% (233/1,151) of cases were >60 years old, and 79.8% (918/1,151) were ≤ 60 years old. When the results of CXRs were set as the reference standard, the sensitivity and specificity in the older group were 0.95(125/132) and 0.85(86/101), respectively, and 0.94(426/453) and 0.93(432/465) in the younger group, respectively (Supplementary Table S1). Nevertheless, the sample size was only 233, which was too small to draw a powerful conclusion.

**Table 4:** Performance of JF CXR-1 against radiologists (PPS).^*^

**Results from JF-CXR1**	**Results from radiologists**		**Total sum**
	**TB (+)**	**TB (–)**	
TB (+)	550	50	600
TB (**–**)	34	516	550
Total sum	584	566	1,150
Sensitivity (95%CI)	94.2% (92.0–95.8%)
Specificity (95%CI)	91.2% (88.5–93.2%)
consistency rate (95%CI)	92.7% (91.1–94.1%)
Kappa	0.854 (*P* = 0.000)

**Table 5:** Performance of JF CXR-1 against final diagnosis (PPS).^*^

**Results from JF CXR-1**	**Results from clinical diagnosis**		**Total sum**
	**TB (+)**	**TB (–)**	
TB (+)	540	42	582
TB (**–**)	141	398	539
Total sum	681	440	1,121
Sensitivity (95%CI)	79.3% (76.1–82.2%)
Specificity (95%CI)	90.5% (87.4–92.9%)
consistency rate (95%CI)	83.7% (81.4–85.7%)
Kappa	0.671 (*P* = 0.000)

* Final diagnosis included bacteriologically confirmed and clinical diagnosed cases.

In the safety set (SS), which included 1,161 participants, 0.3% (3/1,161) had AEs, and no SAE was reported. One had his ankle twisted while running, and two had drug-induced dermatitis after initiating anti-tuberculosis treatment. The AEs were mild, generally not bothersome, and unrelated to the software. No SAE was observed.

## Discussion

In the era of the application of AI, the state-of-the-art in image recognition is CNNs, which have attracted a number of researchers to develop algorithms to replace human readers to identify tuberculosis in chest CXR images (3, 15, 18–24). However, the majority of the software is only tested with retrospective analysis (3, 13, 14, 17, 19, 25–27) or only with datasets (3, 18, 22, 23). Few studies focus on software evaluation in prospective clinical contexts (28, 29). JF CXR-1 v2 had been certified by the National Medical Products Administration of China for the screening and auxiliary diagnosis of active pulmonary tuberculosis in individuals no younger than 15 years and without immunodeficiency. We conducted this prospective multicenter clinical research to evaluate the performance of JF CXR-1 v2 to recognize tuberculosis in persons without immunodeficiency and aged 15 years or older. As described above, 1,151 subjects in the FAS were analyzed. Compared to the results from radiologists on the board (considered the reference standard), the software showed a sensitivity of 94.2% (95% CI: 92.0–95.8%) and a specificity of 91.2% (95% CI: 88.5–93.2%), and the consistency rate was 92.7% (91.1–94.1%) with a Kappa value of 0.854 (*P* = 0.000). The AUC was 0.98 (Figure 3). The results were very close in the PPS. The study of Nijiati et al. (17), which evaluated the performance of a trained AI model with CNNs screening TB in chest CXRs in an underdeveloped area, demonstrated its sensitivity, specificity, consistency rate, and AUC of 85.7%, 94.1%, 91.0%, and 0.910, respectively. Noteworthy, Nijiati et al. (17) also took the results of radiologists as a reference standard because their purpose was screening rather than triage. In a prospective study of a pilot active TB onsite screening project (4), where the reference standard in the project was bacteriologically confirmed, and clinically diagnosed TB, JF CXR-1 had sensitivity, specificity, and AUC of 100.0%, 95.7%, and 0.978 at threshold 30, and of 75.0%, 96.8%, and 0.859 at threshold 50. When the same reference standard was used in our study, the sensitivity, specificity, and consistency rates were 78.9% (75.7–81.8%), 89.9% (86.7–92.4%), and 83.2% (80.9–85.3%) with a Kappa value of 0.66 (*p* = 0.000) in the FAS, and 79.3% (76.1–82.2%), 90.5% (87.4–92.9%), and 83.7% (81.4–85.7%) with a Kappa value of 0.67 (*p* = 0.000) (Table 5) in the PPS.

The first open evaluation of JF CXR-1 was reported by Qin et al. in 2021 (13). JF CXR-1 was one of the five commercial AI algorithms evaluated for TB triaging in Dhaka, Bangladesh, a high-burden setting. Chest CXRs from 23,954 individuals were included in the analysis, and Xpert was set as the reference standard. However, JF CXR-1 has not met the WHO's Target Product Profile (TPP) of a triage test of at least 90.0% sensitivity and at least 70.0% specificity (30), similar to that of InferRead DR and Lunit INSIGHT. It has been proven that JF-CXR-1 significantly outperformed radiologists. When the sensitivity was fixed at 90.0%, the specificity was 61.1% (60.4–61.8%), while when the specificity was fixed at 70.0%, the sensitivity was 85.0% (83.8–86.2%). Moreover, the AUC was 0.849 (0.843–0.855). It was found to reduce half of the required Xpert tests while maintaining a sensitivity above 90.0%. JF CXR-1 had higher sensitivity for most of the decision thresholds (above ~0.15), which confers it more competence to be a better screening tool. Codlin et al. (25) conducted an independent evaluation of CAD software for TB screening; 12 types of software were included. The performance of each software was compared against both an expert and an intermediate human reader. Xpert results were the reference standard, and half of the 12 software programs, including JF CXR-1, achieved similar results on par with the expert reader. The AUC of JF CXR-1 was 0.77 (0.73–0.81), ranking third among the 12 evaluated CAD software.

Currently, there are 17 available or upcoming AI-CAD products for TB detection, including JF CXR-1. Nevertheless, only two are in the catalog of the Global Drug Facility of the Stop TB Partnership: CAD4TB software (The Netherlands) and InferRead DR Chest (Japan). JF CXR-1 is still under evaluation by the WHO (2020 report). CAD4TB is an example in Qin et al.'s retrospective research (13) of comparing the competence of 5 AI-CAD products for TB triaging, including CAD4TB, InferRead DR, Lunit INSIGHT, JF CXR-1, and qXR. CAD4TB was the top performer with sensitivity, specificity, and AUC of 90.0% (89.0–91.0%), 72.9% (72.3–73.5%), and 0.903, respectively. Besides, 49.0% of the people triaged by CAD4TB would be recalled for confirmatory tests in a program focused on capturing almost all people with tuberculosis, while the percentage was 57.0% by JF CXR-1 (13). In another prospective research, (31) which recruited symptomatic adults in a Pakistani hospital and used a reference mycobacterial culture of two sputa, CAD4TB had a sensitivity, specificity, and AUC of 93.0% (90.0–96.0%), 69.0% (67.0–71.0%), and 0.87 (0.85–0.86), respectively. In brief, the AUC of CAD4TB ranges from 0.71 to 0.94 (14, 32), and nearly all the reference standards were set according to the bacteriological results. The sensitivity, specificity, consistency rate, and AUC of JF CXR-1 in our study were non-inferior to most results from other AI-based software. However, since the reference standard in our study was from the human reader instead of bacteriological results, the application of JF CXR-1 is limited to clinical use. While it could play an important role in screening programs, especially in resource-constrained areas (4), the proportion of bacterially confirmed TB only accounts for 63.0% (1). Moreover, the purpose of screening is to pick out people suspected of having TB and those who need further diagnostic evaluation.

Our study has the advantages of a prospective nature and clinical validation, where the CXR data has not been used for software training and testing. There are also limitations. First, participants with obsolete tuberculosis were not included. Considering the confusing presentation of CXRs between obsolete tuberculosis and active tuberculosis, the efficiency of AI software might be lowered once obsolete tuberculosis is included. Second, because our purpose was to evaluate the screening performance of JF CXR-1, the main reference standard was the results drawn from the radiologist rather than the bacteriological results; the bacteriological screen has not been feasible due to financial reasons in a less developed, high-TB-burden country like China. Third, when it comes to real-world use, whether the software performs better than human readers remains uncertain because the comparison to bacteria was not conducted. Fourth, since the tool has shown poor performance in the age group >60 years (13), future studies are needed to fine-tune the algorithm in this area. The technology of AI is used not only for human resource optimization but also for better output. Therefore, we plan to conduct clinical research covering obsolete pulmonary tuberculosis participants and further evaluate the triage performance (with bacterial results) of JF CXR-1 in the future.

Furthermore, since the application of AI in tuberculosis screening is still under exploration, there are some practical considerations, especially in resource-constrained settings. First, some people might have little trust in AI and worry about data safety. Therefore, people undergoing TB screening should be aware that the screening tool utilizes AI software, and they should be given informed consent before proceeding with the screening process. Second, safety should be guaranteed for AI in healthcare (33). Unlike AI-based clinical decision support (CDS) software, screening software might be safer because of diagnosis procedures after screening. However, the reliability, validity, and stability of the software still need to be checked from time to time. Third, the network infrastructure might still be insufficient in resource-constrained settings, and there is the cost of managing network establishment, maintenance, and repair. There are limitations to CNNs: (1) CNNs have high computational requirements, and (2) since the CNNs have multiple layers, the training process takes a particularly long time if the computer does not have a powerful graphics processing unit (GPU). Even if TB screening with AI could save much money, whether resource-constrained areas could afford other costs or not remains problematic.

In our study, the software for tuberculosis screening based on a convolutional neural network algorithm was effective and safe, with satisfying diagnosis performance. It is a potential candidate for solving tuberculosis screening problems in areas lacking radiologists with a high TB burden.

## Data availability statement

The raw data supporting the conclusions of this article will be made available by the authors, without undue reservation.

## Ethics statement

The studies involving humans were approved by The Ethics Committee of Shanghai Public Health Clinical Center, The Ethics Committee of Beijing Chest Hospital, The Ethics Committee of the Third Hospital of Zhenjiang, The Ethics Committee of Chongqing Public Health Medical Center, The Ethics Committee of Jiangxi Province Chest Hospital, The Ethics Committee of Hebei Chest Hospital. The studies were conducted in accordance with the local legislation and institutional requirements. Written informed consent for participation in this study was provided by the participants' legal guardians/next of kin. No potentially identifiable images or data are presented in this study.

## Author contributions

SL designed the study. YY, LX, and PL were responsible for conducting clinical research at Shanghai Public Health Clinical Center. FY, YW, HP, DH, and NL were responsible for conducting research at Chongqing Public Health Medical Center, Jiangxi Chest Hospital, the Third Hospital of Zhenjiang, Beijing Chest Hospital, Hebei Chest Hospital, and respectively. YY and LX gathered the data from all six centers, did the statistical analysis, and wrote the manuscript. All authors contributed to the article and approved the submitted version.

## References

[1] WHO. Global Tuberculosis Report 2022. Geneva: World Health Organization (2022).

[2] WHO Operational Handbook on Tuberculosis. Module 2: Screening - Systematic Screening for Tuberculosis Disease. Geneva: World Health Organizatio. (2021). 33822560

[3] Hwang EJ Park S Jin K-N Kim JI Choi SY Lee JH. Development and validation of a deep learning-based automatic detection algorithm for active pulmonary tuberculosis on chest radiographs. Clin Infect Dis. (2019) 69:739–47. 10.1093/cid/ciy967 30418527 PMC6695514

[4] Liao Q Feng H Li Y Lai X Pan J Zhou F. Evaluation of an artificial intelligence (AI) system to detect tuberculosis on chest X-ray at a pilot active screening project in Guangdong, China in 2019. J Xray Sci Technol. (2022) 30:221–30. 10.3233/XST-211019 34924433 PMC9028657

[5] WHO. Chest Radiography in Tuberculosis Detection: Summary of Current WHO Recommendations and Guidance on Programmatic Approaches. Geneva: World Health Organization (2016). Available online at: https://www.who.int/publications/i/item/9789241511506 (accessed January 3, 2023).

[6] Hoog AHV Meme HK van Deutekom H Mithika AM Olunga C Onyino F. High sensitivity of chest radiograph reading by clinical officers in a tuberculosis prevalence survey. Int J Tuberc Lung Dis. (2011) 15:1308–14. 10.5588/ijtld.11.0004 22283886

[7] Melendez J Sánchez CI Philipsen RHHM Maduskar P Dawson R Theron G. An automated tuberculosis screening strategy combining X-ray-based computer-aided detection and clinical information. Sci Rep. (2016) 6:25265. 10.1038/srep25265 27126741 PMC4850474

[8] Pande T Pai M Khan FA Denkinger CM. Use of chest radiography in the 22 highest tuberculosis burden countries. Eur Respir J. (2015) 46:1816–9. 10.1183/13993003.01064-2015 26405288

[9] Piccazzo R Paparo F Garlaschi G. Diagnostic accuracy of chest radiography for the diagnosis of tuberculosis (TB) and its role in the detection of latent TB infection: a systematic review. J Rheumatol Suppl. (2014) 91:32–40. 10.3899/jrheum.140100 24788998

[10] Pinto LM Pai M Dheda K Schwartzman K Menzies D Steingart KR. Scoring systems using chest radiographic features for the diagnosis of pulmonary tuberculosis in adults: a systematic review. Eur Respir J. (2013) 42:480–94. 10.1183/09031936.00107412 23222871

[11] Van't Hoog A Viney K Biermann O Yang B Leeflang MM Langendam MW. Symptom- and chest-radiography screening for active pulmonary tuberculosis in HIV-negative adults and adults with unknown HIV status. Cochr Database Syst Rev. (2022) 3:CD010890. 10.1002/14651858.CD010890.pub2 35320584 PMC9109771

[12] Cao XF Li Y Xin HN Zhang HR Pai M Gao L. Application of artificial intelligence in digital chest radiography reading for pulmonary tuberculosis screening. Chronic Dis Transl Med. (2021) 7:35–40. 10.1016/j.cdtm.2021.02.001 34013178 PMC8110935

[13] Qin ZZ Ahmed S Sarker MS Paul K Adel ASS Naheyan T. Tuberculosis detection from chest x-rays for triaging in a high tuberculosis-burden setting: an evaluation of five artificial intelligence algorithms. Lancet Digit Health. (2021) 3:e543–54. 10.1016/S2589-7500(21)00116-3 34446265

[14] Qin ZZ Sander MS Rai B Titahong CN Sudrungrot S Laah SN. Using artificial intelligence to read chest radiographs for tuberculosis detection: a multi-site evaluation of the diagnostic accuracy of three deep learning systems. Sci Rep. (2019) 9:15000. 10.1038/s41598-019-51503-3 31628424 PMC6802077

[15] Lakhani P Sundaram B. Deep learning at chest radiography: automated classification of pulmonary tuberculosis by using convolutional neural networks. Radiology. (2017) 284:574–82. 10.1148/radiol.2017162326 28436741

[16] Ma L Wang Y Guo L Zhang Y Wang P Pei X. Developing and verifying automatic detection of active pulmonary tuberculosis from multi-slice spiral CT images based on deep learning. J Xray Sci Technol. (2020) 28:939–51. 10.3233/XST-200662 32651351 PMC12067939

[17] Nijiati M Zhang Z Abulizi A Miao H Tuluhong A Quan S. Deep learning assistance for tuberculosis diagnosis with chest radiography in low-resource settings. J Xray Sci Technol. (2021) 29:785–96. 10.3233/XST-210894 34219703

[18] Nijiati M Ma J Hu C Tuersun A Abulizi A Kelimu A. Artificial intelligence assisting the early detection of active pulmonary tuberculosis from chest X-rays: a population-based study. Front Mol Biosci. (2022) 9:874475. 10.3389/fmolb.2022.874475 35463963 PMC9023793

[19] Lee JH Park S Hwang EJ Goo JM Lee WY Lee S. Deep learning-based automated detection algorithm for active pulmonary tuberculosis on chest radiographs: diagnostic performance in systematic screening of asymptomatic individuals. Eur Radiol. (2021) 31:1069–80. 10.1007/s00330-020-07219-4 32857202

[20] Heo S-J Kim Y Yun S Lim S-S Kim J Nam C-M. Deep learning algorithms with demographic information help to detect tuberculosis in chest radiographs in annual workers' health examination data. Int J Environ Res Public Health. (2019) 16. 10.3390/ijerph16020250 30654560 PMC6352082

[21] Pasa F Golkov V Pfeiffer F Cremers D Pfeiffer D. Efficient deep network architectures for fast chest X-ray tuberculosis screening and visualization. Sci Rep. (2019) 9:6268. 10.1038/s41598-019-42557-4 31000728 PMC6472370

[22] Rajaraman S Zamzmi G Folio LR Antani S. Detecting tuberculosis-consistent findings in lateral chest X-rays using an ensemble of CNNs and vision transformers. Front Genet. (2022) 13:864724. 10.3389/fgene.2022.864724 35281798 PMC8907925

[23] Nafisah SI Muhammad G. Tuberculosis detection in chest radiograph using convolutional neural network architecture and explainable artificial intelligence. Neural Comput Appl. (2022) 19:1–21. 10.1007/s00521-022-07258-6 35462630 PMC9016694

[24] Lee S Yim J-J Kwak N Lee YJ Lee J-K Lee JY. Deep learning to determine the activity of pulmonary tuberculosis on chest radiographs. Radiology. (2021) 301:435–42. 10.1148/radiol.2021210063 34342505

[25] Codlin AJ Dao TP Vo LNQ Forse RJ Van Truong V Dang HM. Independent evaluation of 12 artificial intelligence solutions for the detection of tuberculosis. Sci Rep. (2021) 11:23895. 10.1038/s41598-021-03265-0 34903808 PMC8668935

[26] Tavaziva G Harris M Abidi SK Geric C Breumninger M Dheda K. Chest X-ray analysis with deep learning-based software as a triage test for pulmonary tuberculosis: an individual patient data meta-analysis of diagnostic accuracy. Clin Infect Dis. (2022) 74:1390–400. 10.1093/cid/ciab639 34286831 PMC9049274

[27] Zhou W Cheng G Zhang Z Zhu L Jaeger S Lure FYM. Deep learning-based pulmonary tuberculosis automated detection on chest radiography: large-scale independent testing. Quant Imaging Med Surg. (2022) 12:2344–55. 10.21037/qims-21-676 35371946 PMC8923860

[28] Harris M Qi A Jeagal L Torabi N Menzies D Korobitsyn A. A systematic review of the diagnostic accuracy of artificial intelligence-based computer programs to analyze chest x-rays for pulmonary tuberculosis. PLoS ONE. (2019) 14:e0221339. 10.1371/journal.pone.0221339 31479448 PMC6719854

[29] Tavaziva G Majidulla A Nazish A Saeed S Benedetti A Khan AJ. Diagnostic accuracy of a commercially available, deep learning-based chest X-ray interpretation software for detecting culture-confirmed pulmonary tuberculosis. Int J Infect Dis. (2022) 122:15–20. 10.1016/j.ijid.2022.05.037 35597555

[30] WHO. High Priority Target Product Profiles For New Tuberculosis Diagnostics: Report of a Consensus Meeting. Geneva: World Health Organization (2014). Available online at: https://www.who.int/publications/i/item/WHO-HTM-TB-2014.18 (accessed January 3, 2023).

[31] Khan FA Majidulla A Tavaziva G Nazish A Abidi SK Benedetti A. Chest x-ray analysis with deep learning-based software as a triage test for pulmonary tuberculosis: a prospective study of diagnostic accuracy for culture-confirmed disease. Lancet Digit Health. (2020) 2:e573–81. 10.1016/S2589-7500(20)30221-1 33328086

[32] Kulkarni S Jha S. Artificial intelligence, radiology, and tuberculosis: a review. Acad Radiol. (2020) 27:71–5. 10.1016/j.acra.2019.10.003 31759796

[33] Gerke S Minssen T Cohen G. Chapter 12 - Ethical and legal challenges of artificial intelligence-driven healthcare. Artif Intell Health. (2020) 295–336. 10.1016/B978-0-12-818438-7.00012-5

