# Supplementary material for: A prospective multicenter clinical research study validating the effectiveness and safety of a chest X-ray-based pulmonary tuberculosis screening software JF CXR-1 built on a convolutional neural network algorithm
Source: Front Med (Lausanne). 2023 Aug 15;10:1195451. doi: 10.3389/fmed.2023.1195451 (PMC10463041; doi:10.3389/fmed.2023.1195451)
Supplement: Supplementary file 1 [file Data_Sheet_1.docx]

Table S1 Performance of JF CXR-1 against Radiologists in different age groups（FAS）

| age groups | total | $\frac{AITB (+)}{RadioTB(+)}$ | $\frac{AITB (-)}{RadioTB(-)}$ | sensitivity | specificity |
| --- | --- | --- | --- | --- | --- |
| ≤60 years | 918 | 426/453 | 432/465 | 0.94 | 0.93 |
| >60 years | 233 | 125/132 | 86/101 | 0.95 | 0.85 |

AITB (+): TB positive CXRs determined by AI software, RadioTB (+): TB positive CXRs determined by radiologists, AITB (-): TB negative CXRs determined by AI software, RadioTB (-): TB negative CXRs determined by radiologists.
